# Emergency Department Visit and Rehospitalization in Older Adults Receiving Home Health Nursing: A National Study

**DOI:** 10.1093/geroni/igaf122.2422

**Published:** 2025-12-31

**Authors:** Kayoung Lee, Jeongmin Lee

**Affiliations:** Gachon University, Incheon, Republic of Korea; Seoul National University Hospital, Seoul, Republic of Korea

## Abstract

Older adults with chronic conditions face a high risk of emergency department (ED) visits and rehospitalization after acute hospital discharge. Home health nursing services aim to support safe transitions to home, yet their effectiveness in reducing acute care utilization remains unclear in South Korea. This study analyzes national health insurance claims data from the Health Insurance Review and Assessment Service (HIRA) 2018-2020 to examine home health nursing utilization and factors associated with 30-day rehospitalization and ED visits. Descriptive statistics and logistic regression were used to analyze client characteristics, healthcare utilization, and risk factors. The total number of home health nursing claims increased from 780,911 in 2018 to 1,034,590 in 2019 but declined to 831,947 in 2020, possibly due to COVID-19. The number of clients increased from 90,384 in 2018 to 113,589 in 2019 but decreased to 102,364 in 2020. The average client age was 79.7 years, with over 70% being female. The most common diagnoses varied by facility type, with malignant neoplasms prevalent in tertiary hospitals and mental and behavioral disorders dominant in long-term care hospitals and clinics. ED visits occurred in 0.27% of transitions, while 7.78% of clients were rehospitalized within 30 days. Male sex (OR = 1.28, p < 0.001), older age (OR = 1.02, p < 0.001), and regional disparities significantly influenced readmission risk. The region with the lowest accessibility to healthcare facilities had the highest risk of rehospitalization (OR = 3.23, p < 0.001). Targeted interventions are needed to reduce rehospitalization among high-risk groups and address regional healthcare disparities to improve continuity of care and patient outcomes.

